# Management of cognitive impairment associated with post-COVID-19 syndrome: recommendations for primary care

**DOI:** 10.3389/fphar.2024.1338235

**Published:** 2024-04-22

**Authors:** Udo Zifko, Katja Guendling, Raymond Seet, Siegfried Kasper

**Affiliations:** ^1^ Rudolfinerhaus private clinic GmbH, Rudolfinerhaus, Vienna, Austria; ^2^ Internal Medicine Family Practice, Bad Camberg, Germany; ^3^ Department of Medicine, Yong Loo Lin School of Medicine, National University of Singapore, Singapore, Singapore; ^4^ Center for Brain Research, Department of Molecular Neuroscience, Medical University of Vienna, Vienna, Austria

**Keywords:** post-acute covid, long-COVID syndrome, cognitive impairment, management, Ginkgo biloba extract, EGb 761^®^

## Abstract

**Introduction:** Although post-COVID-19 syndrome (PCS) with cognitive impairment is increasingly encountered in primary care, evidence-based recommendations for its appropriate management are lacking.

**Methods:** A systematic literature search evaluating the diagnosis and treatment of cognitive impairment associated with PCS was conducted. Practical recommendations for the management of PCS-associated cognitive impairment in primary care are summarized, based on an evaluation of pharmacological plausibility and clinical applications.

**Results:** Currently, the pathology of cognitive impairment associated with PCS remains unclear with no high-quality data to support targeted interventions. Existing treatment approaches are directed towards symptom relief where counseling on the chronicity of the disease and regular reassessments at 4- to 8-week intervals is considered reasonable. Patients should be informed and encouraged to adopt a healthy lifestyle that centers around balanced nutrition and appropriate physical activities. They may also benefit from the intake of vitamins, micronutrients, and probiotics. The administration of *Ginkgo biloba* extract could offer a safe and potentially beneficial treatment option. Other non-pharmacological measures include physiotherapy, digitally supported cognitive training, and, if indicated, ergotherapy or speech therapy. In most patients, symptoms improve within 8 weeks. If serious, ambiguous, or when new symptoms occur, specialized diagnostic measures such as comprehensive neurocognitive testing or neuroimaging should be initiated. Very few patients would require inpatient rehabilitation.

**Conclusion:** PCS with cognitive impairment is a debilitating condition that could affect daily functioning and reduce work productivity. Management in primary care should adopt a multidisciplinary approach, centering around physical, cognitive, and pharmacological therapies.

## Introduction

The post-COVID-19 syndrome (PCS, also referred to as long-COVID) is defined by the absence of complete recovery after an acute episode of SARS-CoV-2 infection. According to the clinical case definition published by the World Health Organization (WHO), the post-COVID-19 condition occurs in individuals with a history of probable or confirmed SARS-CoV-2 infection usually 3 months from the onset of COVID-19, with symptoms lasting at least 2 months, and cannot be explained by an alternative diagnosis. Common symptoms include fatigue, shortness of breath, and cognitive dysfunction, which could impact on daily functioning (World Health Organization, 2021). However, there is no widely accepted definition of this condition (Sykes et al., 2021) to date. The prevalence of PCS is estimated at approximately 10% (Siso-Almirall et al., 2021; Davis et al., 2023) of patients infected with SARS-CoV-2, and especially higher among hospitalized patients with COVID-19 pneumonia in excess of 80% (Carfi et al., 2020). Although PCS can develop in patients of all ages, the highest percentage of diagnoses is observed in patients between 36 and 50 years of age, which could impact on work productivity through an increased absence from work (absenteeism) (Davis et al., 2023). A 2021 retrospective cross-sectional study with 1,378 employees undergoing their annual medical check-up in Italy found that PCS was associated with reduced ability to work (Magnavita et al., 2023).

In addition to fatigue, both cognitive impairment and memory complaints in previously cognitively healthy individuals are among the most prominent components in the neurological presentation of PCS. In patients who required hospitalization during SARS- CoV-2 infection, the prevalence of persistent cognitive impairment in the post-acute phase was reported to be 50%–65% (Frontera et al., 2023). According to a meta-analysis of 81 studies, more than one-fifth of patients reported cognitive impairment (Ceban et al., 2022). It is important to note that subjective cognitive dysfunction and objective findings of cognitive impairment post-COVID-19 may be incongruous.

Most patients with persistent symptoms following SARS-CoV-2 infection are managed within the primary care setting. A retrospective data analysis of 63 patients in the COVID-REHA outpatient clinic of the Medical University Hannover in Germany found that only 8% of patients underwent immediate inpatient rehabilitation after first diagnosis (Teixido et al., 2023). An online survey of 11 general practitioners (GPs) in Germany conducted between May and July 2021 revealed that each general practice treated an average of 12 patients with PCS at that time (Schrimpf et al., 2022). In a retrospective cohort analysis of data from health insurance claims for ambulatory care in Bavaria, Germany, between January 2020 and March 2022, 14.2% of patients with confirmed COVID-19 were diagnosed with PCS, and 6.7% received the diagnosis in at least two quarterly periods during a 2-year follow-up (Donnachie et al., 2022). Although the management of PCS-associated cognitive impairment is of utmost importance in primary care, there are no evidence-based guidelines for diagnosis and therapy to date.

The aim of this scoping review was to provide an overview of literature references on PCS-associated cognitive impairment and to reflect the current research perspective. From the outcome combined with personal experience, we aimed to develop pragmatic recommendations with special emphasis on diagnosis and therapy in primary clinical care.

## Materials and methods

A systematic literature search was conducted in PubMed (https://pubmed.ncbi.nlm.nih.gov), covering references published between 1 January 2020 and 7 March 2024 and reporting the management of cognitive impairment associated with PCS (search terms: post-COVID-19 syndrome OR post-acute COVID-19 syndrome OR post-COVID OR long-COVID OR post-COVID-19 condition OR post-acute sequelae of COVID-19 infection OR long-haul COVID OR PSC AND cognitive impairment). By means of additional filters implemented in the PubMed database for the research field COVID-19, the records were further selected electronically for diagnosis (broad scope) and therapy (broad scope). The results were further restricted to English and German language. In addition, a manual search was carried out in the literature known to the authors. The information derived from the publications was summarized and analyzed descriptively.

Key topics of this scoping review include the diagnosis of cognitive impairment associated with PCS and the follow-up of therapeutic outcomes. We thus reviewed publications on cognitive tests, including their sensitivity and specificity for PCS based on published clinical studies, as well as their suitability for the primary care setting.

## Results

**FIGURE 1:**
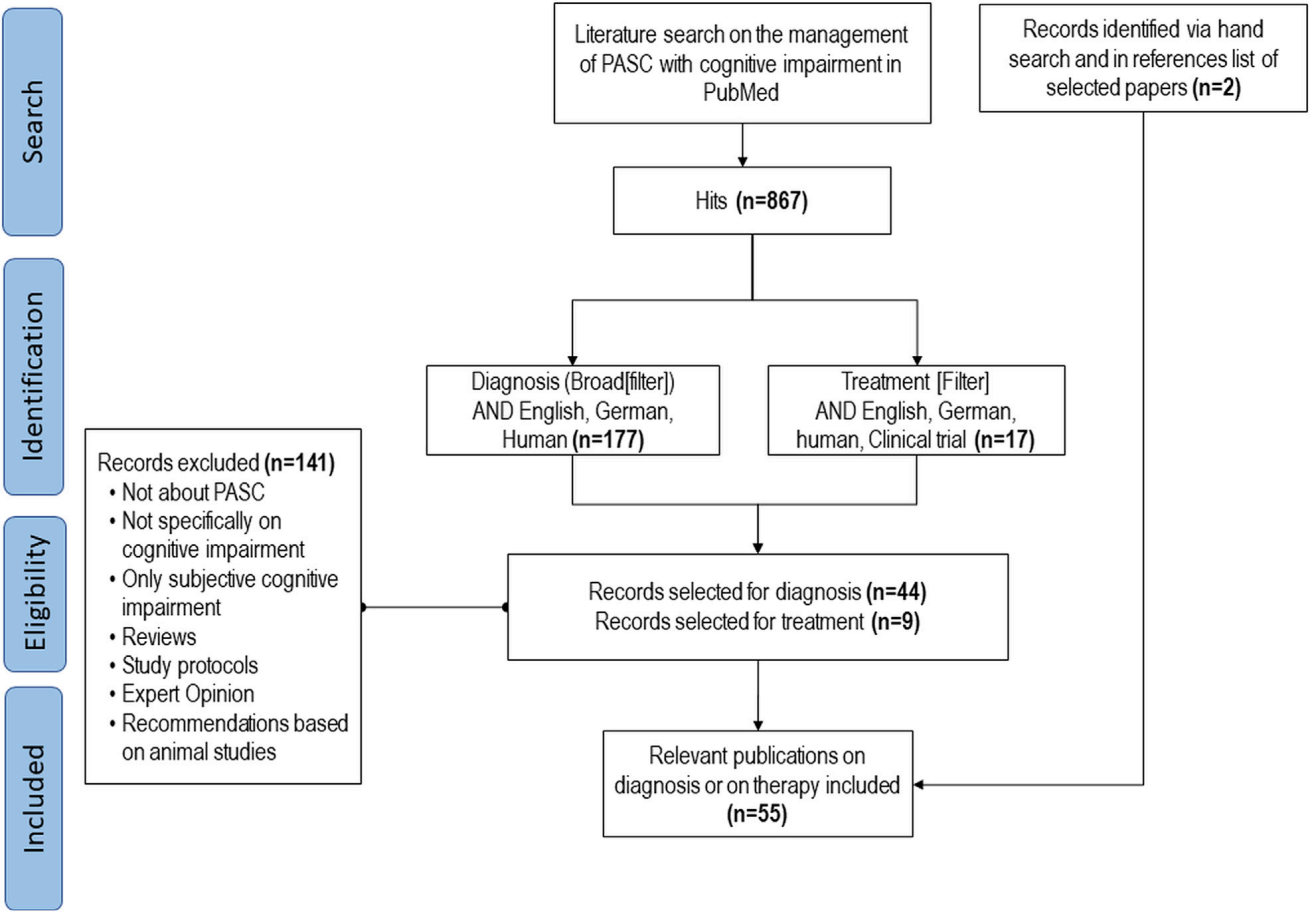
Flow-chart of systematic literature search.

As illustrated in the flow diagram (Figure 1), our literature search resulted in 867 hits. Of these, a total of nine scientific publications were considered as relevant for therapy and 44 for diagnosis of PCS-associated cognitive impairment. Two other publications retrieved by hand search, i.e., the German S1 guideline on long/post-COVID (Rabady et al., 2023) and recommendations for an interdisciplinary and multimodal practical approach for PCS (Teixido et al., 2023), were also included.

### Pathogenesis of cognitive impairment associated with PCS

The pathophysiology underlying PCS remains unclear. Hypotheses on pathogenic mechanisms implicate both systemic effects and sequelae from acute SARS-CoV-2 organ injury. Hyperinflammation (Proal and VanElzakker, 2021), abnormal immunological response and autoimmunity (Leng et al., 2023), viral persistence (Sherif et al., 2023), reactivation of latent Epstein-Barr virus (Su et al., 2022), microvascular dysfunction (Navis, 2023)**,** as well as coagulopathies and endotheliopathy (Leng et al., 2023) have been suggested in the etiopathogenesis of PCS.

Specific pathogenic mechanisms involved in COVID-19-associated cognitive impairment are also not fully understood. The three main factors identified by recent literature reviews (Yang et al., 2021; Monje and Iwasaki, 2022), i.e., neuroinflammation, neurovascular dysfunction, and disruption of cellular energy metabolism, are summarized below. Several other mechanisms are currently being researched, e.g., microbial dysbiosis (Gang et al., 2022), adverse effects of the viral spike protein on the angiotensin-converting enzyme two receptor, or inhibition of the gamma-aminobutyric acid (GABA) receptors (Manganotti et al., 2023).

### Neuroinflammation

As shown by viral detection in the cerebrospinal fluid in some patients, SARS-CoV-2 is neuroinvasive and may spread through various pathways into the central nervous system (Song et al., 2021). This has been suggested to trigger neuroinflammation (Castanares-Zapatero et al., 2022), which may account for neurotoxicity and neuronal damage. Microglia, the resident mononuclear immune cells of the central nervous system (CNS), play an essential role in the response to neuroinflammation. Activated microglia cells in the white matter of the brain further amplify neuroinflammation and can be associated with brain tissue damage (Fernandez-Castaneda et al., 2022). Furthermore, the generation of autoantibodies has a negative impact on neurogenesis and neuronal repair (Elizalde-Diaz et al., 2022).

### Neurovascular dysfunction

Microvascular injury and endothelial damage can trigger excessive thrombin production and inhibit fibrinolysis that leads to formation of microthrombi. These pathogenetic factors may cause hypoperfusion and oxidative stress (Ostergaard, 2021). In a prospective observational cohort study, prolonged endothelial dysfunction with impairments of the microcirculation was observed and may explain ongoing symptoms in PCS (Kuchler et al., 2023).

### Disruption of cellular energy metabolism

Mitochondrial dysfunction, possibly caused by oxidative stress, leads to systemic reduction of metabolic activity and cellular bioenergetics within the CNS structures, which adversely affects neuronal function (Astin et al., 2023; Davis et al., 2023).

### Diagnostic recommendations

**FIGURE 2:**
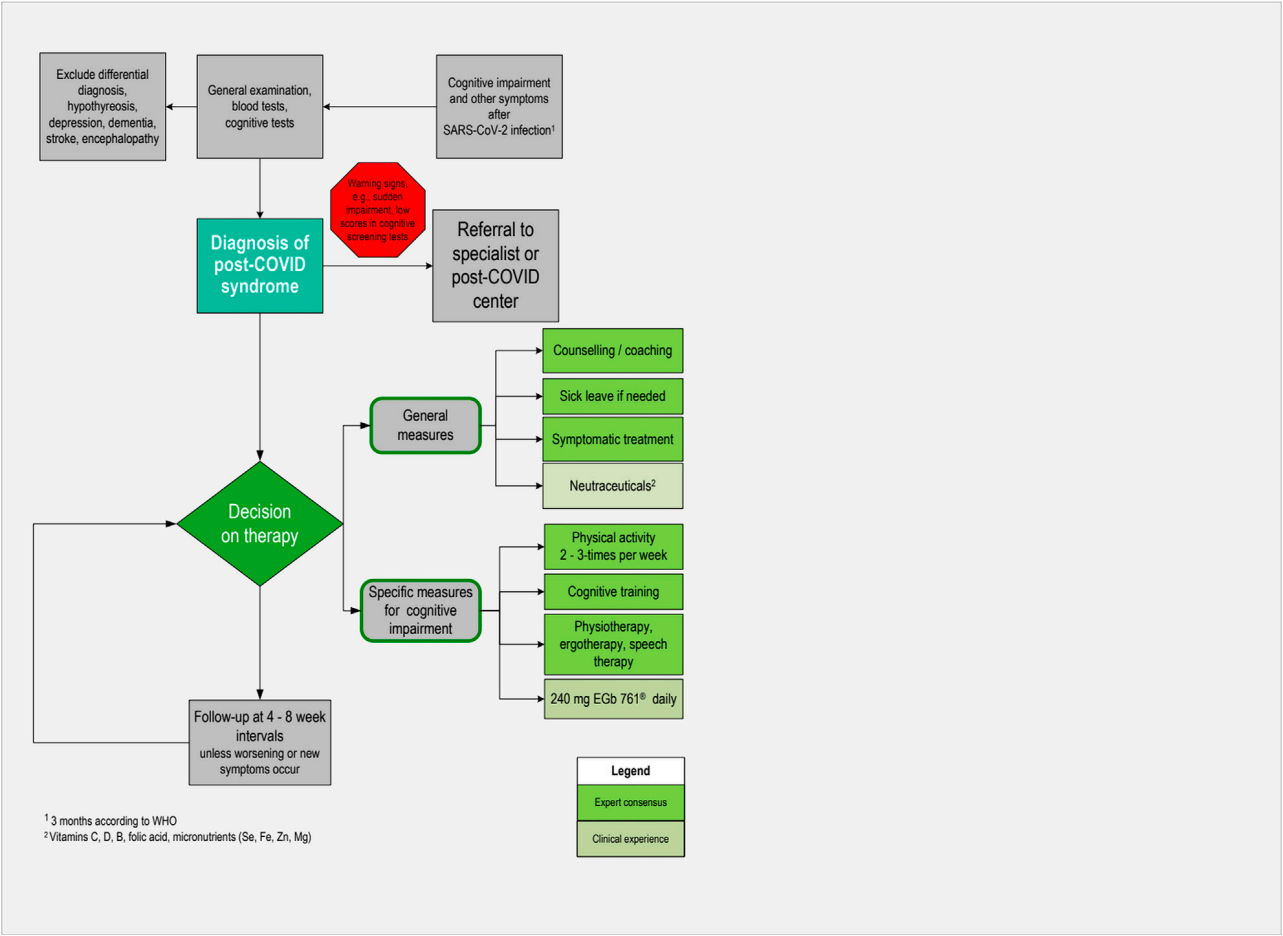
Algorithm for pragmatic management of individuals with cognitive impairment associated with post-COVID syndrome in primary care.

To date, no clear diagnostic criteria or biological markers for PCS have been established. Unless there are warning signs, diagnostics should be handled within the primary care setting. The steps described below have been recommended in a guideline (Koczulla et al., 2022) and are based on clinical experience. An algorithm for pragmatic management of individuals with cognitive impairment associated with post-COVID syndrome in primary care is also depicted in Figure 2.

### History of COVID-19 infection

Firstly, the preceding SARS-CoV-2 infection should be confirmed in patients’ medical history, ideally by polymerase chain reaction (PCR) or by a positive antigen test, even if asymptomatic. In primary care, this should be documented in the relevant patient files. The severity of the preceding infection has relevance for the individual prognosis and can be classified by means of WHO criteria. If patients report cognitive complaints, they should be asked about the onset of symptoms and whether complaints are related to PCS or may have another cause.

### Physical examination

Physical examinations should be tailored to the specific concerns of patients, but should also include assessment of the cardiovascular system and a neurological examination. To assess functional status, the 1-min sit-to-stand test can be used, with the patient sitting down and standing up as often as possible within 1 minute. Depending on their age group, women can perform an average of 27–40 repeats per minute and men 30–45 repeats per minute. Lower values may indicate a reduced physical performance, which then should be examined further. Handgrip strength was shown to be a good indicator for fatigue. It requires a dynamometer and an accurate and standardized conduct.

### Laboratory tests

Blood tests should be performed routinely and include heart, liver, and kidney function parameters. Furthermore, inflammatory markers, i.e., primarily C-reactive protein, immunological parameters, and blood coagulation parameters, should be determined. Testing for COVID-infection markers must be decided case by case, e.g., to exclude an acute infection. In PCS, tests for SARS-CoV-2 have often turned negative when the patient presents.

### Tests of cognitive functioning

Most of the objective cognitive tests applied in clinical studies of PCS were developed in the context of dementia research. As can be seen from the literature found, the domains of cognition that are most frequently impaired in the case of PCS are executive functioning (Calabria et al., 2022; Garcia-Sanchez et al., 2022; Hadad et al., 2022; Serrano-Castro et al., 2022; Shanley et al., 2022; Diez-Cirarda et al., 2023; Ferrucci et al., 2023; Ludwig et al., 2023; Manganotti et al., 2023; Matias-Guiu et al., 2023; Taruffi et al., 2023), attention (Calabria et al., 2022; Garcia-Sanchez et al., 2022; Hadad et al., 2022; Serrano-Castro et al., 2022; Diez-Cirarda et al., 2023; Ferrucci et al., 2023; Ludwig et al., 2023; Matias-Guiu et al., 2023), and delayed recall (Calabria et al., 2022; Crivelli et al., 2022; Garcia-Sanchez et al., 2022; Serrano-Castro et al., 2022; Shanley et al., 2022). Notably, up to two-thirds of patients with subjective cognitive impairment due to PCS still pass these tests successfully, either in the total scores or in single domains (Lauria et al., 2022), which shows the limitations of using these cognitive tests in this patient group. Nevertheless, reliable, short, and easy-to-perform cognitive tests such as the Montreal Cognitive Assessment (MoCA), the Mini-Mental State Examination (MMSE), or the Demenz-Detektion (DemTect) are useful in primary care, especially if their conduct can be delegated to the nursing staff.

Since there is no specific test for PCS-associated cognitive impairment, the MoCA or the MMSE are recommended as global screening tools (Frontera et al., 2023). In 19 of the 44 included studies, the MoCA was applied as an outcome criterion (Nasreddine et al., 2005). Twelve of the studies applied the MMSE. In studies where both tests were applied, the MMSE tended to be less sensitive than the MoCA (Aiello et al., 2022; Schild et al., 2023). Overall, although there are concerns that it may not be sensitive enough to reliably detect mild impairment or impairment in single domains of cognition, MoCA appears to be the most used tool (Lynch et al., 2022). Noteworthy, a positive screening result may indicate a severe course or a comorbid condition requiring special attention while a negative result does not exclude mild cognitive deficits.

Patient self-assessment tools can be used both to identify cognitive impairment and for follow-up (Koczulla et al., 2022). The German S1 guideline on long/post-COVID (Rabady et al., 2023) recommends the abbreviated Measurement of Everyday Cognition (ECog12), which is an assessment tool for self-reported cognitive decline. Another self-assessment tool is the Clinical Global Impression Scale which offers an alternative in terms of a robust measure assessing the global improvement of a patient’s condition. It can be applied to cognition and memory by asking the patient to score from 1 = ‘very much improved’ to 7 = ‘very much worse’. Finally, the five-item version of the Perceived Deficits Questionnaire may be used (Walker et al., 2023), which contains five simple cognition-related questions related to the previous week that are answered by the patients by means of a scale ranging from ‘never’ to ‘almost always’.

### Imaging procedures

In eight studies relating to diagnosis of PCS, imaging procedures such as magnetic resonance imaging (MRI) or computed tomography (CT) were most frequently reported. In a retrospective analysis of case files from 243 patients, 37% were referred for neuroradiological examinations, 31% for electroencephalogram (EEG), and 28% for color Doppler of blood vessels of the head and neck. The results of the tests were not stated (Hegna et al., 2023).

In five studies with patient numbers ranging from 12 to 156, MRI scans of the brain (Franke et al., 2023; Ludwig et al., 2023; Mina et al., 2023; Taruffi et al., 2023), or MRI or CT (Hadad et al., 2022) did not reveal specific pathological findings correlating with cognitive impairment.

From another study including 86 patients with subjective PCS and 36 healthy controls, persistent structural and functional brain abnormalities following MRI examination 11 months after the acute infection were reported (Diez-Cirarda et al., 2023). Bilateral hypometabolism of regions of cerebrum and cerebellum associated with cognitive impairment was detected by positron emission tomography (PET) scan (Guedj et al., 2021) even 1 year after COVID-19 infection (Ferrucci et al., 2023).

### Differential diagnosis

Differential diagnosis is of utmost importance to ensure that cognitive impairment has not been present before the acute phase of COVID-19. The patient, or a caregiver, should be asked since when cognitive problems have existed. Primary care physicians usually have an advantage of knowing the patient’s medical history and thus do not have to rely solely on the patient’s own assessment.

Results from a qualitative study conducted in Germany in primary care provided some insights into how GPs managed patients with PCS (Bachmeier et al., 2023). The exclusion of other underlying conditions, such as hypothyroidism, and other neurologic-psychiatric conditions such as depression, dementia, stroke, or encephalopathy was the most common diagnostic approach.

### Therapeutic recommendations

Since most patients present with moderate or mild symptoms in primary care, a conservative approach including non-pharmacological and pharmacological measures is suitable (Figure 2). According to a practice-based recommendation based on experiences at the COVID-rehabilitation ambulance (Teixido et al., 2023), this includes counseling or coaching of patients and addressing their individual concerns. Besides the restitution of health and capabilities to carry out daily activities, maintaining or regaining the ability to work is of major importance for many patients. They should therefore be informed that complaints due to PCS are mostly reversible within several weeks or months, and usually disappear without any sequelae. It is important for patients to accept that they have to give themselves time for recovery (Bachmeier et al., 2023). Granting sufficiently long sick leave is therefore reasonable in most cases. In a recent study, guided qualitative interviews were conducted with 25 people with PCS (Schmachtenberg et al., 2023). Results showed that many interviewees reached their stress limit during routine household activities or childcare. Of the 25 participants, 19 experienced limitations in pursuing leisure activities, and 10 of those 23 interviewees with jobs reported being on sick leave for several months. Returning to work is possible if daily activities are manageable and 500 m can be walked symptom-free. Work intensity should be discussed with the employer and other restrictions may apply, e.g., avoiding night shifts (Magnavita et al., 2023). Moreover, physicians should discuss with their patients if their cognitive impairment may be a safety issue for professional or leisure activities, particularly for driving or operating machinery.

Follow-up visits at 4- to 8-week intervals are considered appropriate. If patients present with serious, unclear, suddenly evolving symptoms, poor general condition, or other warning signs such as disorientation and confusion, referrals to specialized care are indicated. However, sometimes there are waiting times, particularly for psychotherapy or inpatient rehabilitation.

### Non-pharmacological interventions

General recommendations for patients with cognitive impairment associated with PCS include counseling on lifestyle factors such as improving sleep, reducing stress, adopting a healthy diet, and stopping smoking. A healthy diet consists of ample portions of fruits and salad, prioritizing freshly cooked meals, and reducing meat consumption (Bachmeier et al., 2023). Physical activity plays a crucial role in reducing the impact of PCS and engagement in exercise two to three times a week is recommended (Bachmeier et al., 2023). However, setting realistic and achievable goals and avoiding overexertion is of great importance.

Excessive body weight has been identified as a prognostic factor for poor outcomes of COVID-19. Obesity is likely to impair immune response to viral infections, leading to the development of a chronic low-grade inflammatory state and an elevated level of oxidative stress. Hence, body weight reduction may potentially have a positive effect in obese patients with PCS. In this context, there is ongoing discussion regarding the use of ketogenic diets, which are high-fat diets characterized by a marked carbohydrate restriction. It is important to note that particularly very low-calorie ketogenic diets should be supervised by professionals (Barrea et al., 2022). Implementation of weight reduction training aligns with the goal of strengthening muscles by exercise to improve PCS symptoms and has been found beneficial (Bachmeier et al., 2023; Jimeno-Almazan et al., 2023).

Behavioral interventions mentioned by Müller and Di Benedetto (Muller and Di Benedetto, 2023) comprise mind-body interventions, musical therapy, and meditation. These interventions, which are of low to moderate cost, can be conducted as self-practice at home and can alleviate symptoms of PCS. On a biochemical level, meditation is associated with a release of anti-inflammatory cytokines, modulation of neuroimmune responses, and decrease in C-reactive protein levels.

A practical guideline on PCS including cognitive impairment recommends various non-pharmacological therapeutic measures including digital solutions for cognition or memory training (Teixido et al., 2023). Some of them are freely available on the internet (e.g., for German-speaking patients: https://www.mental-aktiv.de/uebungen-klassisch/) or as YouTube videos with brain training exercises. Others can be prescribed by physicians, e.g., the App NeuroNation MED as digital health application (Digitale Gesundheitsanwendung, DiGA) in Germany. A currently ongoing study is evaluating the efficacy of computer-aided cognitive training in adult patients with PCS (ClinicalTrials.gov ID NCT05338749). Despite the pending results, this study presents an interesting approach to use game-based computer-delivered cognitive training to address mental symptoms such as attention, memory, or deficits in executive functions.

Further elements of outpatient rehabilitation may be physiotherapy, physical rehabilitation, or special fitness measures (Teixido et al., 2023). The RECOVE trial evaluated the effectiveness of exercise and respiratory muscle training administered in outpatient settings for patients with PCS. The authors found that exercises based on concurrent training (including supervised resistance and endurance exercises of low-to-moderate intensity) and concurrent training combined with inspiratory muscle training significantly alleviated dyspnea and fatigue, as well as enhanced overall health status (Jimeno-Almazan et al., 2023).

In specific circumstances, ergotherapy and speech therapy may be recommended. The latter may be necessary if given cognitive communication problems lead to the impairment of language fluency.

### Nutraceuticals

#### Vitamins

The authors of a review article concluded that high-dose intravenous vitamin C (3.5 g–75 g daily) might be a reasonable treatment option for PCS, due to its antioxidant, anti-inflammatory, endothelial-restoring, and immunomodulatory effects (Vollbracht and Kraft, 2021).

Although almost all PCS patients experience vitamin D deficiency, no correlation was found between vitamin D levels and severity of PCS symptoms (Mohamed Hussein et al., 2022). Nevertheless, supplementation of 2,000 IU daily is recommended for patients with vitamin D blood levels below 30 ng/mL (Vieth, 2022).

Vitamins of the B complex are beneficial for the nervous and the immune system. Anecdotal evidence suggests that low-dose vitamin B supplementation (10 mg thiamine, 4 mg riboflavin, 40 mg nicotinamide, 6 mg dexpanthenol daily) improved COVID-19 mortality (Majidi et al., 2022). Dosage recommendations for folic acid supplementation specifically for PSC were not found in the literature. As a general recommendation, vitamins of the B complex should be supplemented unless the blood levels are at the upper limit of the normal range.

### Micronutrients

Micronutrients, including selenium, iron, zinc, and magnesium, are also critical for proper functioning of the immune system. Although there is no evidence for a general benefit in PCS patients, daily supplementation with 35–40 µg selenium, 15 mg iron, 15 mg zinc, or 350 mg magnesium daily can be considered in cases where a deficiency of these nutrients is present (Tosato et al., 2022; Pavlidou et al., 2024).

### Pre- or probiotics

A persisting reduction in the richness of normal composition of gut microbiota can be found even 6 months after recovering from COVID-19 infection. Although controlled clinical trials focusing on patients with PCS are currently lacking, the use of probiotics and prebiotics may be considered as a supportive measure (Catalano et al., 2022). Additionally, immunomodulatory effects of probiotics may help in restoring the gut microbiome altered during viral infections (Muller and Di Benedetto, 2023).

### Pharmacological treatments

To date, the pathologic mechanisms of cognitive impairment associated with PCS are unclear and no evidence-based treatments are available (Bonilla et al., 2023). Thus, management is currently focused on symptomatic treatment including anti-inflammatory drugs such as corticosteroids, anticoagulants, or analgesics, if indicated. The following treatment option specific for cognitive impairment was selected from the literature search based on empirical pharmacological plausibility. Due to the lack of rigorously controlled clinical trials, a favorable safety profile is of high importance.

### Ginkgo biloba extract

Most published nonclinical and clinical studies investigating *Ginkgo biloba* extract were done using the proprietary *Ginkgo biloba* leaves extract EGb 761^®^. EGb 761^®^ was shown to display multimodal effects on a variety of pathogenetic processes which may be involved in PCS (Mueller and Muller, 2024). Flavonoids, terpenoids, and anthocyanidins exhibit neuroprotective effects by modulating signaling pathways known to be impacted by COVID-19 (Zaa et al., 2023). They have been shown to inhibit neuroinflammation by reducing inflammatory activation in microglia cells (Gargouri et al., 2018). Importantly, it protects the function of endothelial cells (Pierre et al., 2008; Zhang et al., 2017) and improves brain and sensory organ perfusion by reducing blood viscosity (Erdinҫler et al., 1996). Randomized controlled trials demonstrated the efficacy of Ginkgo extract at the dose of 240 mg daily in mild cognitive impairment (Grass-Kapanke et al., 2011; Gavrilova et al., 2014). A meta-analysis of seven randomized, placebo-controlled trials in patients with dementia showed that treatment-associated risks (relative risk of adverse events, rates of premature withdrawal) in patients taking EGb 761^®^ did not differ noticeably compared to the placebo group and confirmed the safety and tolerability of this extract (Gauthier and Schlaefke, 2014). However, even if these pharmacological and clinical results are promising, the available data are still preliminary and require additional proof by further studies (Mueller and Muller, 2024).

In patients with cognitive impairment, treatment with Ginkgo extract can be started immediately at first consultation. Follow-up is recommended after 8 weeks of treatment. In a small case series with five patients aged 26–59 years and suffering from concentration and attention deficits, cognitive deficiencies, and/or fatigue, cognitive deficits and other symptoms, such as fatigue and hyposmia, were substantially improved or completely restored by treatment with EGb 761^®^ within 6 months (Zifko et al., 2022). The authors therefore recommended randomized controlled clinical trials to be conducted in order to confirm efficacy in this indication.

### Research perspectives for the management of cognitive impairment associated with PCS

Our literature search revealed nine publications on experimental studies investigating therapeutic approaches. Table 1 presents an overview of study methodologies and main outcomes Table 2. Out of the studies found, only three were small scale randomized controlled studies. Each one was a controlled study on the efficacy of the H2 antagonist famotidine (Momtazmanesh et al., 2023) or donezepil hydrochloride (Pooladgar et al., 2023), and one investigated the effectiveness of a neuro-meditation program (Hausswirth et al., 2023). A case control study evaluated the efficacy of cognitive remediation therapy (Palladini et al., 2023), a feasibility pilot study was carried out on a personalized computerized cognitive training (Dunabeitia et al., 2023), and one observational pilot study evaluated a multimodal therapy concept with behavioral therapy-oriented, disorder-specific psychotherapy and exercise therapy (Kupferschmitt et al., 2023). Moreover, a retrospective analysis was performed with data from 64 patients suffering from PCS who were treated with a day-by-day individualized psychological intervention of cognitive stimulation in addition to a standard in-hospital rehabilitation program (Rabaiotti et al., 2023). A case series with 23 outpatients investigated the effect of transcranial magnetic stimulation (Noda et al., 2023) and another case series reported on five patients treated with EGb 761^®^ following presentation with concentration and attention deficits, cognitive deficiencies, and/or fatigue 9–35 weeks after COVID-19 infection (Zifko et al., 2022).

**TABLE 1:** Data collections on therapy of cognitive impairment associated with PCS.

References	Methodology	Intervention duration	Inclusion criteria	Primary outcomes
Pooladgar et al. (2023)	Randomized, controlled study	5 mg donepezil hydrochloride daily (n = 10) or placebo (n = 15)	Patients with post-COVID memory impairment and a history of hospitalization	The statistical analysis revealed no significant difference in Wechsler Memory Scale-Revised test WMS-R the drug and control groups at the 4-week and 12-week follow-up periods
		12 weeks		
Momtazmanesh et al. (2023)	Randomized, double-blind, placebo controlled study	40 mg famotidine (n = 29) twice daily or placebo (n = 29)	Patients aged 18–65 years with confirmed diagnosis of COVID-19 and Score ≤23 on the Mini-Mental State Examination (MMSE) test or a score ≤22 on the Montreal Cognitive Assessment (MoCA)	The mean difference of the MMSE score of the patients in the famotidine group was significantly higher at week 6 (1.28 (95% CI 0.28–2.28), *p-value* = 0.014)) and at week 12 (2.44 (95% CI 1.47–3.41), *p-value* < 0.001)) compared to those in the placebo group
		12 weeks		
Hausswirth et al. (2023)	Randomized controlled study	Sound therapy and coach-guided meditation associated with light stimulations (n = 17) *versus* no therapy in control group (n = 17) and healthy participants (n = 15)	Patients older than 18 with long COVID (n = 34)	Improvement of cognitive functioning shown by cognitive tests (Choice Response Time, Pattern Comparison Task, Simon Task, Pursuit Rotor Task, Corsi Block-Tapping Task)
		5 weeks	Healthy participants (n = 15)	
Palladini et al. (2023)	Case control study as proof-of-concept study	Cognitive remediation therapy (n = 15) *versus* no therapy (n = 30)	COVID-19 survivors presenting cognitive impairments at 1-month follow-up	Mixed model ANOVA revealed a significant effect over time of the cognitive remediation therapy programme on global cognitive functioning (F = 4.56, *p* = 0.039), while no significant effect was observed in the untreated group
		2 months		
Dunabeitia et al. (2023)	Feasibility Pilot Study with single treatment arm	At least 10 sessions of a self-administered computerized cognitive training with gamified cognitive tasks (n = 73)	Self-reported cognitive dysfunction more than 3 months after a diagnosis of COVID-19	Most of the participants obtained higher scores after computerized cognitive training in each of the domains as compared with baseline
		8 weeks		
Kupferschmitt et al. (2023)	Case documentation	Multimodal therapy concept with behavioural therapy-oriented, disorder-specific psychotherapy and exercise therapy (n = 30)	Patients with post Covid referred to rehabiliation	Positive feed-back of participants on multimodal treatment approach
Rabaiotti et al. (2023)	Observational study	Individualized psychological intervention of cognitive stimulation (45 min) in addition to a standard in-hospital rehabilitation program (n = 64)	Patients with post-acute SARS-CoV-2 cognitive impairment (brain fog)	A significant improvement in the MoCA score was found between admission and discharge (20.4 ± 5vs. 24.7 ± 3.7; *p* < 0.0001)
Noda et al. (2023)	Real word research, case series	20 sessions of transcranial magnetic stimulation treatment (n = 23)	Outpatients with chronic fatigue (n = 12) and cognitive dysfunction (n = 11) associated with long-COVID	The score on the Perceived Deficits Questionnaire–Depression 5-item, which reflects cognitive function, improved from 10.0 to 6.3
		20 days		
Zifko et al. (2022)	Case series	160 mg EGb 761^®^ daily	5 Patients who presented with concentration and attention deficits, cognitive deficiencies, and/or fatigue 9–35 weeks after infection	EGb 761^®^ might be a low-risk treatment option for post-COVID-19 patients with cognitive symptoms
		6–14 weeks		

**TABLE 2:** nine-point WHO ordinal clinical severity scale for COVID-19.

Score	Definition
0	No clinical or neurolgical evidence of infection
1	Ambulatory treatment, no limitation of activities
2	Ambulatory treatment, limitation of activities
3	Hospitalized, no oxygen therapy
4	Hospitalized, oxygen mask or nasal prongs
5	Hospitalized, noninvasive mechanical ventilation or high-flow nasal cannula
6	Hospitalized, intubation and invasive mechanical ventilation
7	Hospitalized, invasive mechanical ventilation and additional support such as pressors or extracardiac membranous oxygenation
8	Death

In August 2023, the US National Library of Medicine of the National Institutes of Health (NIH) clinical research registry (www.clinicaltrials.gov) reported 28 clinical trials investigating treatments of cognitive impairment associated with PCS. Of these, 18 clinical trials investigated various non-pharmacological interventions (ranging from Tai Chi, psychoeducation to computer-based cognitive trainings) and 10 investigated pharmacological treatments (atorvastatin, the NMDA receptor antagonist DAOIB, the antidepressant vortioxetine, an amniotic fluid product VIX001 for intravenous injection, ketamine (CI-581a and b) as glutamatergic modulator, as well as medium chain triglycerides, safflower oil, or nicotinamide riboside as dietary supplements).

## Discussion

PCS-associated cognitive impairment as complication after acute SARS-CoV-2 infection has a negative impact on daily functioning and quality of life and leads to loss of working days or reduced productivity at work and to increased use of healthcare resources. Most patients are managed in a primary care setting requiring a multifactorial and/or multidisciplinary approach with longitudinal follow-up (Siso-Almirall et al., 2021). For cognitive impairment associated with PCS, there are neither clinical guidelines nor well designed randomized controlled trials providing an evidence base for diagnostics and therapy so far. When considering the body of evidence, GPs may feel that they lack sufficient knowledge on this topic. Nevertheless, it is crucial for the healthcare system that only patients requiring secondary or tertiary care are referred to specialists, especially as waiting times amount to several weeks or even months in some countries.

This scoping review provides an overview of literature references on PCS-associated cognitive impairment and develops pragmatic recommendations with special emphasis on diagnosis and therapy in primary clinical care. A similar review of PCS by Nicotra et al. was recently published (Nicotra et al., 2023), which did, however, not focus on primary care. The systematic search resulted in 947 unique records available until May 2023, from which 180 studies were retrieved. The authors stated that only a minority of studies included patients according to stringent temporal criteria for syndrome onset (34%), while most studies reported a required minimum duration of symptoms (77%). In our search, we applied the filters of the literature database for diagnosis and thereby identified 44 clinical studies that applied cognitive tests or imaging procedures (mostly MRI). Nicotra et al. found 36 studies which employed cognitive measures: screening tests alone (n = 19), full neuropsychological batteries (n = 25), or both (n = 29), while 30 studies performed psychiatric testing (Nicotra et al., 2023). Although the numbers vary, the conclusions of the systematic searches are similar. Nicotra et al. reported that cognitive deficits were documented in 39% of subjects, the most frequently affected domains being attention/executive functions (90%) and memory (67%). In our review, only a few individual tests reached positive results in more than 50% of participants. In patients reporting subjective cognitive complaints, measurement based on objective criteria is challenging due to the fact that only half of the patients respond to cognitive tests. In research settings, the conduct of cognitive test batteries is recommended for diagnosis of PCS-associated cognitive impairment. This is time-consuming and not practicable under the conditions of primary care. Since many patients suffer from fatigue, a complete test battery may be too strenuous and is therefore not feasible. Screening tools developed for dementia such as MoCA or MMSE are not sensitive enough for this patient group but are nevertheless useful to identify patients with severe cognitive impairment for whom specialist care is necessary.

Currently, there are many ongoing research activities and clinical studies evaluating PCS. Since there is no uniform definition of the disease, the results are sometimes difficult to interpret. So far, no clearly defined anatomical equivalents or biomarkers for the condition have been found. It seems that most research teams did not find characteristic features in imaging procedures, although this is still an important step in excluding other causes. Moreover, tools for the measurement of cognitive impairment applied in clinical studies are heterogenous and results are therefore not transferrable. The diagnosis or inclusion of patients in almost all studies published relies on self-assessment of patients. All these factors may explain why the results of different studies are equivocal. However, due to the great burden of PCS on the healthcare system, research on the pathogenesis, diagnosis, and therapy is needed. Harmonized methodological approaches are required for future research.

Factors influencing cognitive disturbances in PCS are currently being researched, but the pathogenesis is still not elucidated. Therefore, it is not possible to develop causative drugs that target the condition. Our literature search retrieved only very few publications on clinical studies investigating non-pharmacological or individual pharmacological therapeutic approaches. This is not surprising, given that PCS is a relatively new disease and many studies are therefore still ongoing, as shown by our search in the NIH clinical research registry. While some studies specific to the treatment of PSC-associated cognitive impairment have been published, no confirmatory clinical trials on the efficacy of treatment options or proven therapeutic strategies are available (Frontera et al., 2023). This is due to the relatively new disease and to the fact that there still is a lack of data on the underlying pathophysiological mechanisms. Further research in both fields is therefore needed.

A conservative approach is recommended in primary care unless warning signs such as poor general condition or sudden onset of severe symptoms appear. Pragmatic management strategies consist of a multidisciplinary approach tailored for the individual patient involving counseling on the nature of the disease, optimal lifestyle, digital cognitive training, and non-pharmacological as well as pharmacological therapies.

If indicated, physiotherapy, ergotherapy, or speech therapy can be implemented. Neurocognitive rehabilitation should only be initiated in serious cases, with support from social services (Aiyegbusi et al., 2021). These recommendations may help to allocate resources more efficiently.

Due to its potent anti-inflammatory properties, enhancement of neuroplasticity, and well-proven clinical efficacy, EGb 761^®^ may be beneficial for use in patients with cognitive impairment associated with PCS. In a case series involving patients who experienced persistent cognitive symptoms following SARS-CoV-2 infection, treatment with EGb 761^®^ improved or eliminated cognitive deficits (Zifko et al., 2022). The favorable safety and tolerability profile of EGb 761^®^ supports its use additionally (Schulz et al., 2018). Thus, EGb 761^®^ might be a low-risk treatment option for cognitive impairment associated with PCS.

Symptomatic treatment may include the supplementation of vitamin B complex, vitamin D and micronutrients. Vitamin C may be beneficial due to its antioxidant effect and probiotics have been shown to improve dysbiosis and thereby support the immune system.

Our scoping review may be limited by the fact that no systematic data extraction was carried out and the selection of reports was rather based on subjective assessment of their relevance. Since personal experiences can vary and may not be representative of the broader clinical landscape, the conclusions drawn in our work could be subject to a certain bias. Like all expert recommendations, our findings therefore represent the lowest level of evidence. Nevertheless, we provide a comprehensive overview and analysis of the huge amount of published literature, which might be helpful and time-saving in clinical practice. To our knowledge, this is the first review focusing on primary care as well as on cognitive sequels. Our work also shows that further research is urgently needed to develop evidence-based treatments.

## References

[B1] Aiello E. N. Fiabane E. Manera M. R. Radici A. Grossi F. Ottonello M. (2022). Screening for cognitive sequelae of SARS-CoV-2 infection: a comparison between the mini-mental state examination (MMSE) and the Montreal cognitive assessment (MoCA). Neurol. Sci. 43 (1), 81–84. 10.1007/s10072-021-05630-3 34668124 PMC8526352

[B2] Aiyegbusi O. L. Hughes S. E. Turner G. Rivera S. C. McMullan C. Chandan J. S. (2021). Symptoms, complications and management of long COVID: a review. J. R. Soc. Med. 114 (9), 428–442. 10.1177/01410768211032850 34265229 PMC8450986

[B3] Astin R. Banerjee A. Baker M. R. Dani M. Ford E. Hull J. H. (2023). Long COVID: mechanisms, risk factors and recovery. Exp. Physiol. 108 (1), 12–27. 10.1113/EP090802 36412084 PMC10103775

[B4] Bachmeier B. E. Holzle S. Gasser M. van den Akker M. (2023). How do German general practitioners manage long-/post-COVID? A qualitative study in primary care. Viruses 15 (4), 1016. 10.3390/v15041016 37112996 PMC10146752

[B5] Barrea L. Vetrani C. Caprio M. Cataldi M. Ghoch M. E. Elce A. (2022). From the ketogenic diet to the mediterranean diet: the potential dietary therapy in patients with obesity after CoVID-19 infection (post CoVID syndrome). Curr. Obes. Rep. 11 (3), 144–165. 10.1007/s13679-022-00475-z 35524067 PMC9075143

[B6] Bonilla H. Peluso M. J. Rodgers K. Aberg J. A. Patterson T. F. Tamburro R. (2023). Therapeutic trials for long COVID-19: a call to action from the interventions taskforce of the RECOVER initiative. Front. Immunol. 14, 1129459. 10.3389/fimmu.2023.1129459 36969241 PMC10034329

[B7] Calabria M. Garcia-Sanchez C. Grunden N. Pons C. Arroyo J. A. Gomez-Anson B. (2022). Post-COVID-19 fatigue: the contribution of cognitive and neuropsychiatric symptoms. J. Neurol. 269 (8), 3990–3999. 10.1007/s00415-022-11141-8 35488918 PMC9055007

[B8] Carfi A. Bernabei R. Landi F. Gemelli Against C.-P.-A. C. S. G. (2020). Persistent symptoms in patients after acute COVID-19. JAMA 324 (6), 603–605. 10.1001/jama.2020.12603 32644129 PMC7349096

[B9] Castanares-Zapatero D. Chalon P. Kohn L. Dauvrin M. Detollenaere J. Maertens de Noordhout C. (2022). Pathophysiology and mechanism of long COVID: a comprehensive review. Ann. Med. 54 (1), 1473–1487. 10.1080/07853890.2022.2076901 35594336 PMC9132392

[B10] Catalano A. Iacopetta D. Ceramella J. Maio A. C. Basile G. Giuzio F. (2022). Are nutraceuticals effective in COVID-19 and post-COVID prevention and treatment? Foods 11 (18), 2884. 10.3390/foods11182884 36141012 PMC9498392

[B11] Ceban F. Ling S. Lui L. M. W. Lee Y. Gill H. Teopiz K. M. (2022). Fatigue and cognitive impairment in Post-COVID-19 Syndrome: a systematic review and meta-analysis. Brain Behav. Immun. 101, 93–135. 10.1016/j.bbi.2021.12.020 34973396 PMC8715665

[B12] Crivelli L. Calandri I. Corvalan N. Carello M. A. Keller G. Martinez C. (2022). Cognitive consequences of COVID-19: results of a cohort study from South America. Arq. Neuropsiquiatr. 80 (3), 240–247. 10.1590/0004-282X-ANP-2021-0320 34816972 PMC9648931

[B13] Davis H. E. McCorkell L. Vogel J. M. Topol E. J. (2023). Long COVID: major findings, mechanisms and recommendations. Nat. Rev. Microbiol. 21 (3), 133–146. 10.1038/s41579-022-00846-2 36639608 PMC9839201

[B14] Diez-Cirarda M. Yus M. Gomez-Ruiz N. Polidura C. Gil-Martinez L. Delgado-Alonso C. (2023). Multimodal neuroimaging in post-COVID syndrome and correlation with cognition. Brain 146 (5), 2142–2152. 10.1093/brain/awac384 36288544 PMC9620345

[B15] Donnachie E. Hapfelmeier A. Linde K. Tauscher M. Gerlach R. Greissel A. (2022). Incidence of post-COVID syndrome and associated symptoms in outpatient care in Bavaria, Germany: a retrospective cohort study using routinely collected claims data. BMJ Open 12 (9), e064979. 10.1136/bmjopen-2022-064979 PMC9511014 36137635

[B16] Dunabeitia J. A. Mera F. Baro O. Jadad-Garcia T. Jadad A. R. (2023). Personalized computerized training for cognitive dysfunction after COVID-19: a before-and-after feasibility pilot study. Int. J. Environ. Res. Public Health 20 (4), 3100. 10.3390/ijerph20043100 36833793 PMC9966004

[B17] Elizalde-Diaz J. P. Miranda-Narvaez C. L. Martinez-Lazcano J. C. Martinez-Martinez E. (2022). The relationship between chronic immune response and neurodegenerative damage in long COVID-19. Front. Immunol. 13, 1039427. 10.3389/fimmu.2022.1039427 36591299 PMC9800881

[B18] Erdinҫler D. S. Karakoҫ Y. Toplan S. Önen S. Sukyasyan A. Beğer T. (1996). The effect of ginkgo biloba glycoside on the blood viscosity and erythrocyte deformability. Clin. Hemorheol. 16 (3), 271–276. 10.3233/ch-1996-16306

[B19] Fernandez-Castaneda A. Lu P. Geraghty A. C. Song E. Lee M. H. Wood J. (2022). Mild respiratory COVID can cause multi-lineage neural cell and myelin dysregulation. Cell 185 (14), 2452–2468.e16. 10.1016/j.cell.2022.06.008 35768006 PMC9189143

[B20] Ferrucci R. Cuffaro L. Capozza A. Rosci C. Maiorana N. Groppo E. (2023). Brain positron emission tomography (PET) and cognitive abnormalities one year after COVID-19. J. Neurol. 270 (4), 1823–1834. 10.1007/s00415-022-11543-8 36692636 PMC9873215

[B21] Franke C. Boesl F. Goereci Y. Gerhard A. Schweitzer F. Schroeder M. (2023). Association of cerebrospinal fluid brain-binding autoantibodies with cognitive impairment in post-COVID-19 syndrome. Brain Behav. Immun. 109, 139–143. 10.1016/j.bbi.2023.01.006 36657623 PMC9841734

[B22] Frontera J. A. Guekht A. Allegri R. F. Ashraf M. Baykan B. Crivelli L. (2023). Evaluation and treatment approaches for neurological post-acute sequelae of COVID-19: a consensus statement and scoping review from the global COVID-19 neuro research coalition. J. Neurol. Sci. 454, 120827. 10.1016/j.jns.2023.120827 37856998

[B23] Gang J. Wang H. Xue X. Zhang S. (2022). Microbiota and COVID-19: long-term and complex influencing factors. Front. Microbiol. 13, 963488. 10.3389/fmicb.2022.963488 36033885 PMC9417543

[B24] Garcia-Sanchez C. Calabria M. Grunden N. Pons C. Arroyo J. A. Gomez-Anson B. (2022). Neuropsychological deficits in patients with cognitive complaints after COVID-19. Brain Behav. 12 (3), e2508. 10.1002/brb3.2508 35137561 PMC8933779

[B25] Gargouri B. Carstensen J. Bhatia H. S. Huell M. Dietz G. P. H. Fiebich B. L. (2018). Anti-neuroinflammatory effects of Ginkgo biloba extract EGb761 in LPS-activated primary microglial cells. Phytomedicine 44, 45–55. 10.1016/j.phymed.2018.04.009 29895492

[B26] Gauthier S. Schlaefke S. (2014). Efficacy and tolerability of Ginkgo biloba extract EGb 761^®^ in dementia: a systematic review and meta-analysis of randomized placebo-controlled trials. Clin. Interv. Aging 9, 2065–2077. 10.2147/CIA.S72728 25506211 PMC4259871

[B27] Gavrilova S. I. Preuss U. W. Wong J. W. Hoerr R. Kaschel R. Bachinskaya N. (2014). Efficacy and safety of Ginkgo biloba extract EGb 761 in mild cognitive impairment with neuropsychiatric symptoms: a randomized, placebo-controlled, double-blind, multi-center trial. Int. J. Geriatr. Psychiatry 29 (10), 1087–1095. 10.1002/gps.4103 24633934

[B28] Grass-Kapanke B. Busmane A. Lasmanis A. Hoerr R. Kaschel R. (2011). Effects of ginkgo biloba special extract EGb 761&amp;#174; in very mild cognitive impairment (vMCI). Neurosci. Med. 2 (01), 48–56. 10.4236/nm.2011.21007

[B29] Guedj E. Campion J. Y. Dudouet P. Kaphan E. Bregeon F. Tissot-Dupont H. (2021). (18)F-FDG brain PET hypometabolism in patients with long COVID. Eur. J. Nucl. Med. Mol. Imaging 48 (9), 2823–2833. 10.1007/s00259-021-05215-4 33501506 PMC7837643

[B30] Hadad R. Khoury J. Stanger C. Fisher T. Schneer S. Ben-Hayun R. (2022). Cognitive dysfunction following COVID-19 infection. J. Neurovirol 28 (3), 430–437. 10.1007/s13365-022-01079-y 35618983 PMC9134977

[B31] Hausswirth C. Schmit C. Rougier Y. Coste A. (2023). Positive impacts of a four-week neuro-meditation program on cognitive function in post-acute sequelae of COVID-19 patients: a randomized controlled trial. Int. J. Environ. Res. Public Health 20 (2), 1361. 10.3390/ijerph20021361 36674117 PMC9858974

[B32] Hegna E. Racki V. Hero M. Papic E. Rozmaric G. Radovic K. (2023). Post-COVID-19 syndrome in neurology patients: a single center experience. Pathogens 12 (6), 796. 10.3390/pathogens12060796 37375486 PMC10302491

[B33] Jimeno-Almazan A. Buendia-Romero A. Martinez-Cava A. Franco-Lopez F. Sanchez-Alcaraz B. J. Courel-Ibanez J. (2023). Effects of a concurrent training, respiratory muscle exercise, and self-management recommendations on recovery from post-COVID-19 conditions: the RECOVE trial. J. Appl. Physiol. 134 (1), 95–104. 10.1152/japplphysiol.00489.2022 36476156 PMC9829459

[B34] Koczulla A. R. Ankermann T. Behrends U. Berlit P. Berner R. Böing S. (2022). AWMF S1-leitlinie long/post-COVID (S1 guideline long/post-COVID). Available at: https://register.awmf.org/de/leitlinien/detail/020-027 (Accessed August, 2023). 10.1055/a-1946-3230 36479679

[B35] Kuchler T. Gunthner R. Ribeiro A. Hausinger R. Streese L. Wohnl A. (2023). Persistent endothelial dysfunction in post-COVID-19 syndrome and its associations with symptom severity and chronic inflammation. Angiogenesis 26, 547–563. 10.1007/s10456-023-09885-6 37507580 PMC10542303

[B36] Kupferschmitt A. Etzrodt F. Kleinschmidt J. Kollner V. (2023). Not only multimodal, but also interdisciplinary: a concept for interdisciplinary cooperation in the rehabilitation of post-COVID syndrome. Psychother. Psychosom. Med. Psychol. 73 (1), 34–41. 10.1055/a-1838-3055 35605967

[B37] Lauria A. Carfi A. Benvenuto F. Bramato G. Ciciarello F. Rocchi S. (2022). Neuropsychological measures of long COVID-19 fog in older subjects. Clin. Geriatr. Med. 38 (3), 593–603. 10.1016/j.cger.2022.05.003 35868675 PMC9080120

[B38] Leng A. Shah M. Ahmad S. A. Premraj L. Wildi K. Li Bassi G. (2023). Pathogenesis underlying neurological manifestations of long COVID syndrome and potential therapeutics. Cells 12 (5), 816. 10.3390/cells12050816 36899952 PMC10001044

[B39] Ludwig B. Deckert M. Krajnc N. Keritam O. Macher S. Bsteh G. (2023). Reported neurological symptoms after severe acute respiratory syndrome coronavirus type 2 infection: a systematic diagnostic approach. Eur. J. Neurol. 30 (9), 2713–2725. 10.1111/ene.15923 37306533

[B40] Lynch S. Ferrando S. J. Dornbush R. Shahar S. Smiley A. Klepacz L. (2022). Screening for brain fog: is the montreal cognitive assessment an effective screening tool for neurocognitive complaints post-COVID-19? Gen. Hosp. Psychiatry 78, 80–86. 10.1016/j.genhosppsych.2022.07.013 35930974 PMC9359801

[B41] Magnavita N. Arnesano G. Di Prinzio R. R. Gasbarri M. Meraglia I. Merella M. (2023). Post-COVID symptoms in occupational cohorts: effects on health and work ability. Int. J. Environ. Res. Public Health 20 (9), 5638. 10.3390/ijerph20095638 37174158 PMC10178744

[B42] Majidi N. Bahadori E. Shekari S. Gholamalizadeh M. Tajadod S. Ajami M. (2022). Effects of supplementation with low-dose group B vitamins on clinical and biochemical parameters in critically ill patients with COVID-19: a randomized clinical trial. Expert Rev. Anti Infect. Ther., 1–7. 10.1080/14787210.2022.2125867 36108676

[B43] Manganotti P. Michelutti M. Furlanis G. Deodato M. Buoite Stella A. (2023). Deficient GABABergic and glutamatergic excitability in the motor cortex of patients with long-COVID and cognitive impairment. Clin. Neurophysiol. 151, 83–91. 10.1016/j.clinph.2023.04.010 37210757 PMC10170904

[B44] Matias-Guiu J. A. Herrera E. Gonzalez-Nosti M. Krishnan K. Delgado-Alonso C. Diez-Cirarda M. (2023). Development of criteria for cognitive dysfunction in post-COVID syndrome: the IC-CoDi-COVID approach. Psychiatry Res. 319, 115006. 10.1016/j.psychres.2022.115006 36521337

[B45] Mina Y. Enose-Akahata Y. Hammoud D. A. Videckis A. J. Narpala S. R. O'Connell S. E. (2023). Deep phenotyping of neurologic postacute sequelae of SARS-CoV-2 infection. Neurol. Neuroimmunol. Neuroinflamm 10 (4), e200097. 10.1212/NXI.0000000000200097 37147136 PMC10162706

[B46] Mohamed Hussein A. A. R. Galal I. Amin M. T. Moshnib A. A. Makhlouf N. A. Makhlouf H. A. (2022). Prevalence of vitamin D deficiency among patients attending Post COVID-19 follow-up clinic: a cross-sectional study. Eur. Rev. Med. Pharmacol. Sci. 26 (8), 3038–3045. 10.26355/eurrev_202204_28635 35503606

[B47] Momtazmanesh S. Ansari S. Izadi Z. Shobeiri P. Vatankhah V. Seifi A. (2023). Effect of famotidine on cognitive and behavioral dysfunctions induced in post-COVID-19 infection: a randomized, double-blind, and placebo-controlled study. J. Psychosom. Res. 172, 111389. 10.1016/j.jpsychores.2023.111389 37327698 PMC10229204

[B48] Monje M. Iwasaki A. (2022). The neurobiology of long COVID. Neuron 110 (21), 3484–3496. 10.1016/j.neuron.2022.10.006 36288726 PMC9537254

[B49] Mueller J. K. Muller W. E. (2024). Multi-target drugs for the treatment of cognitive impairment and fatigue in post-COVID syndrome: focus on Ginkgo biloba and Rhodiola rosea. J. Neural Transm. (Vienna) 131 (3), 203–212. 10.1007/s00702-024-02749-3 38347175 PMC10874325

[B50] Muller L. Di Benedetto S. (2023). Aged brain and neuroimmune responses to COVID-19: post-acute sequelae and modulatory effects of behavioral and nutritional interventions. Immun. Ageing 20 (1), 17. 10.1186/s12979-023-00341-z 37046272 PMC10090758

[B51] Nasreddine Z. S. Phillips N. A. Bedirian V. Charbonneau S. Whitehead V. Collin I. (2005). The Montreal Cognitive Assessment, MoCA: a brief screening tool for mild cognitive impairment. J. Am. Geriatr. Soc. 53 (4), 695–699. 10.1111/j.1532-5415.2005.53221.x 15817019

[B52] Navis A. (2023). A review of neurological symptoms in long COVID and clinical management. Semin. Neurol. 43 (2), 286–296. 10.1055/s-0043-1767781 37068519

[B53] Nicotra A. Masserini F. Calcaterra F. Di Vito C. Doneddu P. E. Pomati S. (2023). What do we mean by long-COVID? A scoping review of the cognitive sequelae of SARS-CoV-2 infection. Eur. J. Neurol. 30, 3968–3978. 10.1111/ene.16027 37540896

[B54] Noda Y. Sato A. Shichi M. Sato A. Fujii K. Iwasa M. (2023). Real world research on transcranial magnetic stimulation treatment strategies for neuropsychiatric symptoms with long-COVID in Japan. Asian J. Psychiatr. 81, 103438. 10.1016/j.ajp.2022.103438 36610206 PMC9795803

[B55] Ostergaard L. (2021). SARS CoV-2 related microvascular damage and symptoms during and after COVID-19: consequences of capillary transit-time changes, tissue hypoxia and inflammation. Physiol. Rep. 9 (3), e14726. 10.14814/phy2.14726 33523608 PMC7849453

[B56] Palladini M. Bravi B. Colombo F. Caselani E. Di Pasquasio C. D'Orsi G. (2023). Cognitive remediation therapy for post-acute persistent cognitive deficits in COVID-19 survivors: a proof-of-concept study. Neuropsychol. Rehabil. 33 (7), 1207–1224. 10.1080/09602011.2022.2075016 35583357

[B57] Pavlidou E. Poulios E. Papadopoulou S. K. Fasoulas A. Dakanalis A. Giaginis C. (2024). Clinical evidence on the potential beneficial effects of diet and dietary supplements against COVID-19 infection risk and symptoms' severity. Med. Sci. (Basel) 12 (1), 11. 10.3390/medsci12010011 38390861 PMC10885051

[B58] Pierre S. V. Lesnik P. Moreau M. Bonello L. Droy-Lefaix M. T. Sennoune S. (2008). The standardized Ginkgo biloba extract Egb-761 protects vascular endothelium exposed to oxidized low density lipoproteins. Cell Mol. Biol. (Noisy-le-grand) 54, OL1032–1042. 18954552

[B59] Pooladgar P. Sakhabakhsh M. Soleiman-Meigooni S. Taghva A. Nasiri M. Darazam I. A. (2023). The effect of donepezil hydrochloride on post-COVID memory impairment: a randomized controlled trial. J. Clin. Neurosci. 118, 168–174. 10.1016/j.jocn.2023.09.005 37952347

[B60] Proal A. D. VanElzakker M. B. (2021). Long COVID or post-acute sequelae of COVID-19 (PASC): an overview of biological factors that may contribute to persistent symptoms. Front. Microbiol. 12, 698169. 10.3389/fmicb.2021.698169 34248921 PMC8260991

[B61] Rabady S. Hoffmann K. Aigner M. Altenberger J. Brose M. Costa U. (2023). S1 guidelines for the management of postviral conditions using the example of post-COVID-19. Wien Klin. Wochenschr 135 (4), 525–598. 10.1007/s00508-023-02242-z 37555900 PMC10504206

[B62] Rabaiotti P. Ciraci C. Donelli D. Oggioni C. Rizzi B. Savi F. (2023). Effects of multidisciplinary rehabilitation enhanced with neuropsychological treatment on post-acute SARS-CoV-2 cognitive impairment (brain fog): an observational study. Brain Sci. 13 (5), 791. 10.3390/brainsci13050791 37239263 PMC10216015

[B63] Schild A. K. Goereci Y. Scharfenberg D. Klein K. Lulling J. Meiberth D. (2023). Multidomain cognitive impairment in non-hospitalized patients with the post-COVID-19 syndrome: results from a prospective monocentric cohort. J. Neurol. 270 (3), 1215–1223. 10.1007/s00415-022-11444-w 36422669 PMC9686246

[B64] Schmachtenberg T. Muller F. Kranz J. Dragaqina A. Wegener G. Konigs G. (2023). How do long COVID patients perceive their current life situation and occupational perspective? Results of a qualitative interview study in Germany. Front. Public Health 11, 1155193. 10.3389/fpubh.2023.1155193 36969629 PMC10034079

[B65] Schrimpf A. Braesigk A. Lippmann S. Bleckwenn M. (2022). Management and treatment of long COVID symptoms in general practices: an online-based survey. Front. Public Health 10, 937100. 10.3389/fpubh.2022.937100 36176520 PMC9513068

[B66] Schulz M. Hoerr R. Mueller H. (2018). 46th ESCP symposium on clinical pharmacy "Science meets practice: towards evidence-based clinical pharmacy services", Heidelberg, Germany, October 9th-11th, 2017. Int. J. Clin. Pharm. 40 (1), 203–317. 10.1007/s11096-017-0565-9 29222732

[B67] Serrano-Castro P. J. Garzon-Maldonado F. J. Casado-Naranjo I. Ollero-Ortiz A. Minguez-Castellanos A. Iglesias-Espinosa M. (2022). The cognitive and psychiatric subacute impairment in severe Covid-19. Sci. Rep. 12 (1), 3563. 10.1038/s41598-022-07559-9 35241761 PMC8894467

[B68] Shanley J. E. Valenciano A. F. Timmons G. Miner A. E. Kakarla V. Rempe T. (2022). Longitudinal evaluation of neurologic-post acute sequelae SARS-CoV-2 infection symptoms. Ann. Clin. Transl. Neurol. 9 (7), 995–1010. 10.1002/acn3.51578 35702954 PMC9268882

[B69] Sherif Z. A. Gomez C. R. Connors T. J. Henrich T. J. Reeves W. B. Force R. M. P. T. (2023). Pathogenic mechanisms of post-acute sequelae of SARS-CoV-2 infection (PASC). Elife 12, e86002. 10.7554/eLife.86002 36947108 PMC10032659

[B70] Siso-Almirall A. Brito-Zeron P. Conangla Ferrin L. Kostov B. Moragas Moreno A. Mestres J. (2021). Long covid-19: proposed primary care clinical guidelines for diagnosis and disease management. Int. J. Environ. Res. Public Health 18 (8), 4350. 10.3390/ijerph18084350 33923972 PMC8073248

[B71] Song E. Zhang C. Israelow B. Lu-Culligan A. Prado A. V. Skriabine S. (2021). Neuroinvasion of SARS-CoV-2 in human and mouse brain. J. Exp. Med. 218 (3), e20202135. 10.1084/jem.20202135 33433624 PMC7808299

[B72] Su Y. Yuan D. Chen D. G. Ng R. H. Wang K. Choi J. (2022). Multiple early factors anticipate post-acute COVID-19 sequelae. Cell 185 (5), 881–895.e20. 10.1016/j.cell.2022.01.014 35216672 PMC8786632

[B73] Sykes D. L. Holdsworth L. Jawad N. Gunasekera P. Morice A. H. Crooks M. G. (2021). Post-COVID-19 symptom burden: what is long-COVID and how should we manage it? Lung 199 (2), 113–119. 10.1007/s00408-021-00423-z 33569660 PMC7875681

[B74] Taruffi L. Muccioli L. Mitolo M. Ferri L. Descovich C. Mazzoni S. (2023). Neurological manifestations of long COVID: a single-center one-year experience. Neuropsychiatr. Dis. Treat. 19, 311–319. 10.2147/NDT.S387501 36761395 PMC9904212

[B75] Teixido L. Andreeva E. Gartmann J. Lemhofer C. Sturm C. Gutenbrunner C. (2023). Outpatient rehabilitative care for patients with Long-COVID - a guideline-based clinical practice guideline. Laryngorhinootologie 102 (7), 521–532. 10.1055/a-1985-0450 37130538

[B76] Tosato M. Ciciarello F. Zazzara M. B. Pais C. Savera G. Picca A. (2022). Nutraceuticals and dietary supplements for older adults with long COVID-19. Clin. Geriatr. Med. 38 (3), 565–591. 10.1016/j.cger.2022.04.004 35868674 PMC9212635

[B77] Vieth R. (2022). Critique of public health guidance for vitamin D and sun exposure in the context of cancer and COVID-19. Anticancer Res. 42 (10), 5027–5034. 10.21873/anticanres.16011 36191997

[B78] Vollbracht C. Kraft K. (2021). Feasibility of vitamin C in the treatment of post viral fatigue with focus on long COVID, based on a systematic review of IV vitamin C on fatigue. Nutrients 13 (4), 1154. 10.3390/nu13041154 33807280 PMC8066596

[B79] Walker S. Goodfellow H. Pookarnjanamorakot P. Murray E. Bindman J. Blandford A. (2023). Impact of fatigue as the primary determinant of functional limitations among patients with post-COVID-19 syndrome: a cross-sectional observational study. BMJ Open 13 (6), e069217. 10.1136/bmjopen-2022-069217 PMC10335413 37286327

[B80] World Health Organization (2021). A clinical case definition of post COVID-19 condition by a Delphi consensus, 6 October 2021. Geneva: World Health Organization.

[B81] Yang F. Zhao H. Liu H. Wu X. Li Y. (2021). Manifestations and mechanisms of central nervous system damage caused by SARS-CoV-2. Brain Res. Bull. 177, 155–163. 10.1016/j.brainresbull.2021.09.015 34571039 PMC8462004

[B82] Zaa C. A. Espitia C. Reyes-Barrera K. L. An Z. Velasco-Velazquez M. A. (2023). Neuroprotective agents with therapeutic potential for COVID-19. Biomolecules 13 (11), 1585. 10.3390/biom13111585 38002267 PMC10669388

[B83] Zhang C. Wang D. F. Zhang Z. Han D. Yang K. (2017). EGb 761 protects cardiac microvascular endothelial cells against hypoxia/reoxygenation injury and exerts inhibitory effect on the ATM pathway. J. Microbiol. Biotechnol. 27 (3), 584–590. 10.4014/jmb.1611.11024 27974731

[B84] Zifko U. A. Yacob M. Braun B. J. Dietz G. P. H. (2022). Alleviation of post-COVID-19 cognitive deficits by treatment with EGb 761^®^: a case series. Am. J. Case Rep. 23, e937094. 10.12659/AJCR.937094 36156538 PMC9523733

